# Replacement of Native with Malted Triticale (x *Triticosecale* Wittmack) Flour in Dry Pasta: Technological and Nutritional Implications

**DOI:** 10.3390/foods13152315

**Published:** 2024-07-23

**Authors:** Mariasole Cervini, Chiara Lobuono, Federica Volpe, Francesco Matteo Curatolo, Francesca Scazzina, Margherita Dall’Asta, Gianluca Giuberti

**Affiliations:** 1 Department for Sustainable Food Process, Università Cattolica del Sacro Cuore, 29122 Piacenza, Italy; mariasole.cervini@unicatt.it (M.C.); gianluca.giuberti@unicatt.it (G.G.); 2 Department of Food and Drug, University of Parma, 43125 Parma, Italy; chiara.lobuono@unipr.it (C.L.); francesca.scazzina@unipr.it (F.S.); 3 Department of Animal Science, Food and Nutrition, Università Cattolica del Sacro Cuore, 29122 Piacenza, Italy; federica.volpe@unicatt.it (F.V.); francescomatteo.curatolo@unicatt.it (F.M.C.)

**Keywords:** triticale, extrusion, in vitro starch digestion, in vitro protein digestion

## Abstract

The use of native and malted triticale (MT) flour in dry pasta has been limited despite the potential of triticale in cereal-based food production. In this study, triticale-based dry spaghetti with increasing levels of substitution (0, 25, 50, and 75 g/100 g *w*/*w*) of MT flour were formulated and analyzed. Samples were analyzed for technological and nutritional traits, including the in vitro starch and protein digestions. The gradual substitution of native triticale flour with MT increased (*p* < 0.05) the total dietary fiber content, whereas total starch decreased (*p* < 0.05). Adding MT flour increased the cooking loss and the stickiness of cooked pasta (*p* < 0.05). Using MT flour modulated the in vitro starch digestion, lowering the slowly digestible and resistant starch contents. The in vitro protein digestibility was positively affected using MT at the highest substitution level. Overall, MT could be used to formulate dry pasta products being the substitution to native triticale up to 50 g/100 g, a good compromise between nutritional quality and technological characteristics.

## 1. Introduction

Triticale (x *Triticosecale* Wittmack) is a man-made cereal derived from the hybridization of wheat species with rye, aiming to join wheat’s functional characteristics for food production with rye’s tolerance to non-optimal growing conditions [1,2]. The current triticale production has risen to over 20 million tonnes per year and is mainly concentrated in Europe, producing more than 80% of the world’s total [1]. Even if a wide variation has been reported, the nutritional composition of whole grain triticale flour is more comparable to wheat than rye. On average, triticale flour is characterized by a relatively high content of starch (>70 g/100 g), protein (>13 g/100 g), and dietary fiber (>14 g/100 g) [1]. The interest in utilizing triticale flour for different food applications in the food market is growing, aiming to introduce new/rediscovered cereals to complement wheat varieties [2]. Overall, several studies confirmed the potential of using triticale for cereal-based food production by selecting suitable genotypes and using feasible processing/formulation conditions [1,2]. Bread, biscuits, tortillas, noodles, and fresh pasta have been formulated with triticale flour alone or in combination with gluten-containing and gluten-free flours, reporting encouraging results [1,2,3]. For instance, it has been shown that blending triticale flour (up to 70% *w*/*w*) with wheat flour can contribute to formulating bread with promising nutritional composition and quality, comparable to wheat flour only [4]. However, few studies on the in vitro digestibility of dietary components, including starch and protein, in triticale-containing foods are present in the literature [1,5]. Among the different processing conditions, the malting process, also known as sprouting or germination, is gaining interest as an effective and economical biological process. A typical malting process consists of three main steps: (i) steeping to hydrate and reactivate the resting grains’ metabolism; (ii) controlled germination, during which the grain undergoes several chemical and structural modifications; (iii) kilning to stop metabolic processes and ensure a shelf-stable ingredient [6]. Several studies have indicated that malting/germination can promote an improvement in the nutritional value of the grains, as recently reviewed [7,8,9]. The nutritional benefits include (i) enhanced digestibility and bioavailability of nutrients; (ii) generation of new bioactive compounds; (iii) lower levels of anti-nutrients, such as enzyme inhibitors and metal-chelating species. In addition to the nutritional components, the malting process can make processed grains more palatable [9]. In starch-based products, such as bread, it has been reported that germinated wheat flour can improve the product’s structural qualities, such as softness and crumb structure [10]. In addition, malted sorghum flour has been used in place of up to 60% *w*/*w* of wheat flour in gluten-containing biscuits without remarkable changes in technological quality and sensory attributes [7]. Nevertheless, germination can cause a reduction in gluten strength and protein weakening in the mixing properties, such as farinography values, mixolab parameters, and pasting properties [7]. Differences in malting conditions, types of cereals, levels of inclusion of malted flours in the recipe, types of food products, and methodology might account for contrasting results inherent to the technological performance of the selected malted flours [11,12]. The nutritional and quality effects of native and malted triticale flours in dry pasta formulation have not been thoroughly investigated. This may be of concern considering the trend in the food market to formulate starch-based products with unconventional and under-exploited flours. Considering that native triticale grains are a promising cereal for malting due to the high levels of α-amylase and proteolytic enzymes [9], this study aimed to formulate a novel dry pasta containing only native triticale flour and increasing levels of malted triticale (MT) flour (up to 75% *w*/*w*) as a replacement for the native counterpart.

To the best of our knowledge, there are no studies in which triticale flours (native and malted) were employed in dry pasta formulation. Therefore, this study aimed to formulate dry pasta with increasing level of malted triticale flour in the recipe, aiming to explore the suitability of triticale flour (native and malted) for this type of food product. Long pasta was specifically produced for the study and analyzed considering technological and nutritional characteristics, including the evaluation of the in vitro starch and protein digestibility.

## 2. Materials and Methods

### 2.1. Raw Materials and Ingredient Preparation

Whole triticale grains were commercially available. As reported on the label, the nutritional composition (100 g of product) was 13.3 g protein, 2.1 total lipids, 64.3 g total carbohydrates, and 12.4 g total dietary fiber. Whole grains were treated in an automatic Micromalting System (Phoenix Biosystems, Adelaide, Australia) as detailed by Piazza et al. [5]. In brief, the malting process lasted for 144 h, consisting of 14 subsequent steps (i.e., 1 step of cleaning, 3 steps of steeping, 5 steps of germination, and 5 steps of kilning) with a temperature range from 15 to 80 °C. At the end of the malting process, rootlets and coleoptiles were manually removed. Native and malted triticale grains were then milled (particle size < 300 μm; Ultra Centrifugal Mill ZM 300, Retsch, Haan, Germany).

### 2.2. Pasta Preparation

Whole grain triticale flour was replaced with 0, 25, 50, and 75 g/100 g *w*/*w* of MT flour to obtain control (CTR), 25-MT, 50-MT, and 75-MT pasta samples, respectively. A RZ50 pasta machine was used (La Parmigiana, Fidenza, Italy). Flour blends and tap water at 38 °C were horizontally mixed (11 min) to obtain a dough with a total moisture content of about 35 g/100 g. A 1.7 mm bronze spaghetti-shaped die was used. The temperature during the extrusion step was kept <50 °C, and the auger screw extrusion speed was 20 rpm. Spaghetti was cut 20 cm long and dried with a low-temperature drying cycle at 50 °C for 12 h (La Parmigiana ESS20, Fidenza, Italy). For each recipe, three batches were produced. The highest substitution level of 75 g/100 g of MT to native triticale flour was identified considering preliminary extrusion tests. Going beyond this level caused structural deformation of pasta shape during the extrusion process.

### 2.3. Chemical Composition of Pasta

The chemical composition of malted flour and raw pasta samples was assessed through AOAC official methods of analysis [13]. Total starch was measured using an enzymatic assay kit (K-TSTA-100A; Megazyme Europe Ltd.; Ayr, Scotland, UK). The total dietary fiber (TDF), soluble dietary fiber (SDF), and insoluble dietary fiber (IDF) contents were assessed through the enzyme kit INTDF02/15 (Megazyme Europe Ltd.; Ayr, Scotland, UK). Free sugar content (sum of sucrose, D-glucose, and D-fructose) was measured using the enzyme kit K-SUFRG (Megazyme Europe Ltd.; Ayr, Scotland, UK).

### 2.4. Pasta Quality, Texture, and Thermal Properties

The optimum cooking time (OCT) was evaluated with the AACC-approved method 66.50 [14] by cooking 25 g of pasta in boiling distilled water at 1:10 pasta–water. Cooking loss (CL) and water absorption capacity (WAC) were determined through standard methods of analysis (AACC 66-50) [14]. Cooked pasta texture as maximum cutting force (i.e., firmeness; AACC method 66-50; AACC 2011) was evaluated with a TA-XT2i Texture Analyser (Stable Micro Systems, Godalming, UK; 5 kg load cell; light knife blade A/LKB; and a speed of 0.17 mm/s). Six pasta strands were used for the analysis. A pasta firmness/stickiness rig (HDP/PFS) was employed to evaluate the surface stickiness with a compression speed of 0.5 mm/s and a compression force of 1 kg for 2 s. Ten measurements for each sample were performed. The thermal properties of raw pasta samples were determined by differential scanning calorimetry (DSC) (DSC8000, Perkin Elmer Inc., Waltham, MA, USA) [15]. A 1:3 *w*/*w* ground sample–distilled water ratio was used. After 20 h of equilibration at room temperature, samples were heated from 25 to 180 °C at 10 °C/min. The onset temperature (To), the peak temperature (Tp), the conclusion temperature (Tc), and the gelatinization enthalpy (∆H; as J/g dry starch) were recorded. Four measurements for each sample were conducted. The surface color of uncooked samples and samples cooked to optimum was measured using a Minolta CR410 Chroma Meter (Konica Minolta Co., Tokyo, Japan) and the resulting color parameters (i.e., L*, a*, and b*) were used to calculate the total color differences (∆E*) before and after cooking to CTR [15].

### 2.5. In Vitro Starch and Protein Digestion of Cooked Pasta

Samples cooked to optimum underwent an in vitro starch digestion (IVSD) protocol according to Englyst et al. [16]. After cooking, 20 g of each sample was minced in an automatic meat grinder (7 mm holes; Adler Ad 4808, Adler Europe Group, Warsaw, Poland). Then, an aliquot of 2 g was carefully weighed into plastic tubes and 10 mL of pepsin-guar solution (5 g/L pepsin P7000, Sigma-Aldrich, St. Louis, MO, USA) and 5 g/L guar (G4129, Sigma-Aldrich, St. Louis, MO, USA) in 0.05 M HCl) were added. The IVSD was started by incubation at 37 °C for 30 min under horizontal agitation (180 rpm). Five glass marbles and 10 mL of 0.25 M sodium acetate (37 °C) were added. Five mL of an enzyme mixture was then added to each sample, and samples were incubated at 37 °C and 200 rpm. The enzyme mixture was prepared according to Dodi et al. [17] using pancreatin (P7545, Sigma-Aldrich, St. Louis, MO, USA), amyloglucosidase (A7095, Sigma-Aldrich, St. Louis, MO, USA), and invertase (I4504, Sigma-Aldrich, St. Louis, MO, USA). After 20 min and 120 min, liquid aliquots (1 mL) were collected, centrifuged (14,000 rpm for 5 min), and the supernatant was diluted in distilled water (ratio 1:10), and used to determine the total glucose concentration (TG). The glucose released after 20 min of incubation (G20) and 120 min of incubation (G120) was analyzed colorimetrically (GODPOD 4058, Giesse Diagnostic snc, Rome, Italy). Values were then used to calculate the amount of rapidly digestible starch (RDS) and slowly digestible starch (SDS), as detailed by Dodi et al. [17]. Available starch (AS) was calculated as the sum of RDS and SDS. In addition, the SDS/AS ratio was calculated on cooked pasta based on the SDS and AS contents. For each treatment, batches were analyzed in quadruplicate. The resistant starch (RS) content in cooked pasta samples was assessed enzymatically (K-RSTAR 02/17, Megazyme Europe Ltd.; Scotland, UK). The in vitro protein digestibility (IVPD) of cooked pasta samples was evaluated as detailed by Suo et al. [18] using enzyme hydrolysis at 37 °C based on pepsin (P7000, Merck KGaA, Darmstadt, Germany) and pancreatin (P7545, Merck KGaA, Darmstadt, Germany) enzymes. The IVPD was calculated as a percentage of the total nitrogen content of samples before the enzyme hydrolysis and after correction for blank.

### 2.6. Statistical Analysis

The normal distribution of data was determined using the Shapiro–Wilk test before statistical analysis. Data were analyzed as a completely randomized design using the GLM procedure of SAS 9.3 (SAS Inst. Inc., Cary, NC, USA) according to the model: Yij = μ + αi + eij, where Yij is the dependent variable on the jth subject (pasta batch) assigned to treatment i, μ is the overall mean, αi is the fixed effect of MT flour substitution level to native triticale flour, and eij is the residual error. Significance was declared at *p* < 0.05.

## 3. Results and Discussion

### 3.1. Changes in the Chemical Composition of Flour and Pasta Samples

The applied malting process numerically modified the chemical composition of flour. When compared to the native counterpart, the MT flour was characterized by similar levels of moisture (10.3 versus 10.2 g water/100 g of food), protein (13.3 versus 13.5 g/100 g of flour), and lipid (2.1 versus 2.0 g/100 g of flour), respectively. On the contrary, numerically lower total starch (57.9 versus 64.3 g/100 g of flour) and numerically greater TDF (15.8 versus 12.4 g/100 g of flour) contents were measured by comparing MT to native flour, respectively. In addition, numerically greater free sugars (about +55%) were measured in MT flour compared to the native counterpart, confirming previous findings [12]. Several physicochemical changes can modify the grain’s chemical and nutritional composition during the malting process. Starch is partially hydrolyzed to mono- and disaccharides by the action of amylases, whereas the composition of amino acids may be altered because of the action of protease enzymes, even if the total protein content may be unchanged [7,9]. In particular, the malting process can promote starch’s enzymatic breakdown into simple sugars by activating endogenous enzymes such as α- and β-amylases and α-glucosidase [19]. In addition, the increase in the TDF content can be related to the cleavage of intermolecular bonds, and the increase in the cellular structure of the grains during germination [20]. Comparable changes following a malting step of grains have been reported previously [5,20]. As expected, the gradual substitution of native triticale flour with MT flour changed the nutritional composition of spaghetti (Table 1).

Accordingly, total starch decreased (*p* < 0.05) from 62.2 to 58.1 g/100 g dry pasta, whereas the TDF and the free sugar contents increased (*p* < 0.05) from 10.3 to 14.3 g/100 g dry pasta and from 1.8 to 2.7 g/100 g dry pasta with increasing levels of MT flour in the recipe, respectively. In addition, the SDF/IDF ratio increased (*p* < 0.05) with increasing substitution levels of MT flour in spaghetti formulation, with the highest values recorded for 50-MT and 75-MT samples. All other nutritional parameters were not influenced (*p* > 0.05) when comparing the different formulations. These changes reflected the different nutritional composition of triticale flour before and after the malting process and the degree of substitution in the recipe [5]. Various studies reported an increase in the TDF and changes in the total carbohydrate content following germination as a function of the inherent process conditions [21]. For instance, an increase in the soluble dietary fiber fraction has been reported in germinated wheat flour as compared to the control [21]. Following the malting of finger millet for 96 h, the total starch content decreased from 65 to 43 g/100 g dry matter [22]. Germinated wheat flour increased bread’s total free sugar content to about 60% at the highest inclusion level [10].

The increase in dietary fiber content in MT-based products is positive since it may contribute to the overall daily dietary fiber intake and thus promote different benefits for human health, such as the positive modulation of the gut microbiota composition, lower concentrations of serum inflammatory biomarkers, and a lower risk of coronary heart disease [23,24].

### 3.2. Pasta Cooking Quality, and Texture, Thermal, and Surface Color Properties of Spaghetti

Native triticale-based pasta had the longest OTC, at 7.6 min (*p* < 0.05) (Table 2). Increasing the level of MT flour in the recipe as a replacement for native triticale flour reduced (*p* < 0.05) the OCT, with the lowest value recorded for 75-MT spaghetti (i.e., 6.7 min, *p* < 0.05). Fuad and Prabhasankar [25] indicated that the starch granules enzymatically broken-down during germination may provide faster gelatinization on cooking, thus resulting in shorter OCT of pasta.

The CL for good-quality pasta should not exceed the threshold value of 8 g/100 g of product [15]. In this study, the incorporation of MT flour contributed to gradually increasing (*p* < 0.05) the CL, thus indicating more solids to be released into the cooking water. However, the recorded values were all below 8%, which can be considered satisfactory. Similar trends in the OCT and CL were reported by Xing et al. [26] for pasta formulated with germinated quinoa flour and by Kömürcü and Bilgiçli [27] using germinated ancient wheat flour in noodles. The WAC of pasta decreased with the addition of MT flour, with the lowest value recorded for 75-MT (i.e., 109.3%; *p* < 0.05). It has been suggested that the WAC was positively related to the starch content in pasta [26]. In addition, shorter cooking time can correspond to lower WAC due to less starch granule hydration [15].

Texture parameters of cooked spaghetti are reported in Table 2. The firmness values of spaghetti formulated with increasing levels of MT flour in the recipe were lower (*p* < 0.05) than CTR spaghetti. In particular, the lowest value was recorded for 75-MT (i.e., 5.04 N; *p* < 0.05). As gluten quality is mainly responsible for the quality of pasta with good textural characteristics, the decrease in the firmness values as the level of MT flour increased in the formulation may be due to the weakening of the gluten network because of increased protease activity during the germination process [28]. In particular, the breakdown of gluten during germination primarily occurs because of peptide bond hydrolysis, followed by the disruption of secondary bonds (i.e., ionic, hydrogen, and hydrophobic bonds), which are known to play a role in the physical structure of gluten [11]. In line with the current findings, Liu et al. [29] observed a decrease in the firmness of cooked wheat-based noodles substituted with different proportions of sprouted mung bean flour in the recipe. The firmness reduction may also be related to the dilution of gluten, owing to the high fiber content of MT flour [28,30]. Similar results were obtained by Manthey and Schorno [31], who reported lower firmness values of spaghetti formulated with whole wheat flour. In addition, the decrease in pasta firmness agrees with the increase in CL values, which aligns with previous findings [15].

High-quality cooked pasta should have minimal surface stickiness values [32]. As reported in Table 2, stickiness increased as the level of MT flour increased in pasta formulation, with the highest value obtained at the substitution level of 75 g/100 g of MT to native triticale flour (i.e., 3.5 N; *p* < 0.05). In general, the higher the CL, the stickier the pasta [32]. Pasta stickiness is related to constituents (mainly amylose and amylopectin leached from the gelatinized starch granules) escaping from the protein network and adhering to the surface of cooked pasta [33]. The increase in stickiness as a function of the inclusion level of MT flour in the recipe might indicate a progressive weakening of the gluten network, which contributes to reducing the gluten network’s ability to wrap the starch, thus increasing its leaching [28,30].

The To, Tp, Tc, and ∆H mean values of native triticale-based pasta were 60.1, 66.4, and 72.2 °C and 5.8 J/g dry starch, respectively (Table 2). Namir et al. [34] reported that 100% wheat pasta was characterized by endothermic transitions between 55 and 72 °C, which aligns with the current findings. By increasing the substitution level of native triticale flour with MT flour in pasta formulation, To and Tp gradually increased (*p* < 0.05) from 60.1 to 63.1 °C and from 66.4 to 68.1 °C, while Tc and ∆H gradually decreased (*p* < 0.05) from 72.2 to 70.5 °C and from 5.8 to 5.1 J/g dry starch, for CTR and 75-MT pasta, respectively. These changes indicated that endotherms became shorter as a function of the MT flour level in the recipe and peaks occurred at higher temperatures. Similar changes in the thermal transition temperatures have been reported by Jribi et al. [35] evaluating the thermal properties of sprouted whole durum wheat flour and by Li et al. [36] in germinated brown rice starch. These changes might be linked to structural changes in starch occurring during germination. For instance, Oseguera-Toledo et al. [37] showed that sorghum starch granules undergo a transformation during malting, increasing the superficial pores due to the enzymatic activity. Furthermore, the germination process is known to contribute to hydrolyzing starch. According to Xu et al. [38], a decrease in ∆H after germination can suggest that less energy is required to break down intermolecular hydrogen bonds of starch granules.

Pasta color is an essential factor in assessing pasta quality. The average values of the L*, a*, and b* coordinates are presented in Table 3. In general, pasta containing MT flour had lower lightness (L*) and greater yellowness and redness (b* and a*) values than the CTR pasta (*p* < 0.05), irrespective of the level of substitution and the cooking process. Therefore, increasing the level of MT flour in the recipe enhanced the dark, red, and yellow tint of spaghetti. Similar color changes have been reported in gluten-free fresh egg spaghetti formulated with malted pulses [39]. It has been reported that color changes during malting are strictly dependent on both enzymatic and non-enzymatic browning reactions, including the initial oxidation of polyphenol compounds and the accumulation of melanoidin formed during the reaction of low molecular sugars and proteins [40,41]. In addition, the cooking process altered the color of spaghetti (Table 3). Comparing the color coordinates before and after cooking, an increase in L* and a decrease in a* and b* parameters can be observed, irrespective of the level of inclusion of MT flour in the recipe. This could be due to a loss of water-soluble vegetable pigments during cooking. However, before and after cooking, all the MT flour-containing spaghetti was characterized by ∆E* values > 3 to the CTR, meaning that the human eye could easily distinguish these samples.

### 3.3. In Vitro Starch and Protein Digestion of Cooked Pasta

**Figure 1:**
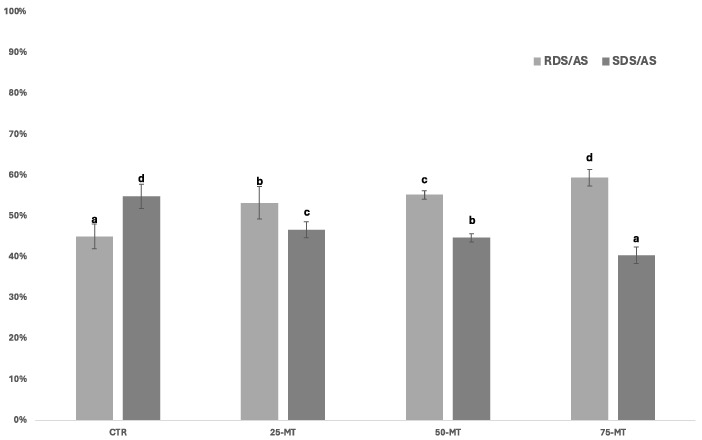
Rapidly and slowly digestible starch (RDS and SDS) expressed as percentages of the available starch (AS; mean ± SD) for the pasta samples. CTR: dry pasta formulated with 0:100 *w*/*w* malted triticale (MT) flour–native triticale flour; 25-MT: dry pasta formulated with 25:75 *w*/*w* MT flour–native triticale flour; 50-MT: dry pasta formulated with 50:50 *w*/*w* MT flour–native triticale flour; 75-MT: dry pasta formulated with 75:25 *w*/*w* MT flour–native triticale flour. Values with different letters within the same metric indicate statistical significance (*p* < 0.05). For each recipe, reported values represented the average of three pasta batches.

According to Englyst et al.’s [16] classification, starch in foods can be categorized into three fractions: RDS, SDS, and RS. The RDS is the fraction of starch digested within 20 min that is likely available for rapid absorption in the small intestine, whereas SDS is the fraction that can be wholly and slowly digested up to 120 min, thus contributing to favorably modulating the glycemic response [42]. Instead, the RS fraction refers to starch that arrives unaltered in the colon, where it can be modified and metabolized by the gut microbiota, and it may act as a prebiotic [43]. Scientific evidence highlighted the association between SDS content in starchy foods and the reduction in postprandial glucose release into the bloodstream [44,45]. In particular, to meet the health claim related to “slowly digestible starch in starch-containing foods” and “reduction of postprandial glycemic responses”, at least 40% of the total available starch in a certain food should be in the form of SDS [44]. As reported in Table 4, spaghetti formulated with 100% native triticale flour (CTR) exhibited the highest SDS content (i.e., 16.3 g/100 g of product as eaten; *p* < 0.05) and the lowest RDS content (i.e., 13.4 g/100 g of product as eaten; *p* < 0.05) with respect to MT-containing samples. In general, gluten-containing pasta is recognized as one of the primary sources of SDS within starch-rich foods [46,47], with an RS content ranging from 1.5 to 3.4 g/100 g of food [48]. Our findings support these indications, thus suggesting that native triticale flour can be used in pasta formulation to obtain a product with good nutritional characteristics in terms of in vitro starch digestion. Increasing the substitution level of MT flour in spaghetti was reflected in a different content of starch fractions; the RDS content gradually increased (*p* < 0.05), whereas the SDS content decreased due to the inclusion of MT flour (*p* < 0.05). The RS content was found to be the highest in CTR pasta (i.e., 3.0 g/100 g of product as eaten; *p* < 0.05), while the lowest was found in 50- and 75-MT samples (i.e., on average 2.6 g/100 g of product as eaten; *p* < 0.05). In addition, the RDS/AS ratio increased (*p* < 0.05), whereas the SDS/AS progressively decreased (*p* < 0.05) as the level of MT flour increased in spaghetti formulation, but it remained > 40% for all of the experimental samples (Figure 1).

Contrasting results concerning the effect of germination/malting on the IVSD are present in the literature. Marti et al. [10] evidenced decreased IVSD in wheat bread formulated with germinated flour. In contrast, the studies of Yang et al. [20] and Piazza et al. [5] showed that germination/malting contributed to enhancing the IVSD of different cereal flours and cereal-based food products. In addition, the germination process may induce a reduction in the relative percentage of RS and an increase in the relative percentage of the total available starch content [49]. Differences in the selected starting plant material, germination/malting duration and conditions, and in vitro methods can explain current discrepancies. Present data suggested that a possible drawback of the adopted malting process can be that the inherent starch structure can be partially degraded by the action of the endogenous enzyme hydrolysis, making it easier for starch to be degraded by the action of starch-hydrolyzing enzymes, thus modulating the starch fraction content [20]. Moreover, the increased fiber content of the MT-enriched pasta samples may also negatively affect starch digestibility, as previously described [47]. Gallo et al. [50] described that fiber caused an undesirable increase in the RDS value while lowering the SDS content for commercial whole wheat spaghetti compared to the traditional ones. Since the glycemic response appears to be strongly related to the rapidly and slowly digestible starch fraction contained in starchy foods [42,45], further investigations are recommended to optimize the adopted malting conditions to modulate the IVSD in related food products positively.

The IVPD of samples after cooking is reported in Table 4. In all cases, the IVPD was closer to or higher than 90%, with the 75-MT pasta showing the highest values among samples (i.e., 92.7%; *p* < 0.05). It has been reported that germination/malting can improve the IVPD in native cereal and legume flours, due to the activation of proteolysis, lowering the levels of anti-nutrient protease inhibitors, activating proteases, and breaking down storage proteins [9,51]. For instance, Sharma and Gujral [52] reported that the IVPD of raw finger and proso millet increased from 72 to 84% and from 75 to 83% during 48 h of germination. The current increase in IVPD following the use of MT flour in spaghetti was numerically lower compared to the data reported in the literature on raw vegetable materials [51]. Presumably, under the current experimental conditions, the inherent effect of malting on the IVPD of spaghetti may have been masked by the pasta processing conditions (i.e., extrusion and drying) along with the cooking phase, these being recognized as important factors that can mutually contribute to enhancing the IVPD in different food products [53,54].

## 4. Conclusions

The present study confirmed the suitability of native triticale flour in dry pasta formulation and that the use of malted triticale flour can contribute to increasing the total dietary fiber content, while decreasing the total starch. Overall, significant differences in pasta quality parameters were detected. Spaghetti becomes softer and stickier when the level of MT increases in the formulation, thus suggesting a progressive weakening of the gluten network. Moreover, the inclusion of MT increased the digestibility of starch compared to the 100% native triticale pasta, even though the SDS/available starch fraction was still significant in 75-MT pasta. It can also be observed that the MT substitution from 25 to 50% *w*/*w* may represent a good compromise between nutritional quality and technological characteristics of dry pasta. Further investigations, including a sensory evaluation of samples, are recommended to optimize the adopted triticale grain malting conditions to limit possible drawbacks in quality parameters while maintaining, if not improving, the overall nutritional quality.

## Figures and Tables

**Table 1:** Nutritional composition (g/100 g food) of spaghetti formulated with increasing levels of malted triticale (MT) flour.

	Substitution with MT				√MSE
Parameters (g/100 g)	CTR ^1^	25-MT ^2^	50-MT ^3^	75-MT ^4^	
Moisture	10.2 ^a^	10.1 ^a^	10.1 ^a^	10.2 ^a^	0.12
Total starch	62.2 ^d^	60.9 ^c^	59.7 ^b^	58.1 ^a^	1.43
Sugars	1.8 ^a^	2.1 ^ab^	2.3 ^b^	2.7 ^c^	0.31
Protein	12.8 ^a^	12.9 ^a^	13.0 ^a^	13.1 ^a^	0.96
Lipid	1.9 ^a^	2.0 ^a^	2.0 ^a^	2.0 ^a^	0.24
Total dietary fiber	9.3 ^a^	11.7 ^b^	13.0 ^c^	14.3 ^d^	2.11
Soluble dietary fiber	3.4 ^a^	4.1 ^b^	4.9 ^c^	5.6 ^d^	1.08
Insoluble dietary fiber	5.9 ^a^	7.6 ^b^	8.1 ^c^	8.6 ^d^	1.45
Ash	0.9 ^a^	0.9 ^a^	1.0 ^a^	0.9 ^a^	0.03

Values with different superscripts within the same line differed at *p* < 0.05. For each recipe, reported values represented the average of the three pasta batches. ^1^ Dry pasta formulated with 0:100 *w*/*w* MT flour–native triticale flour. ^2^ Dry pasta formulated with 25:75 *w*/*w* MT flour–native triticale flour. ^3^ Dry pasta formulated with 50:50 *w*/*w* MT flour–native triticale flour. ^4^ Dry pasta formulated with 75:25 *w*/*w* MT flour–native triticale flour.

**Table 2:** Quality parameters and starch thermal properties of spaghetti formulated with increasing levels of malted triticale (MT) flour.

	Substitution with MT				√MSE
	CTR ^1^	25-MT ^2^	50-MT ^3^	75-MT ^4^	
*Pasta quality*					
Optimum cooking time (min)	7.6 ^d^	7.3 ^c^	6.9 ^b^	6.7 ^a^	1.12
Water absorption capacity (%)	158.4 ^d^	149.3 ^c^	122.4 ^b^	109.3 ^a^	7.43
Cooking loss (%)	6.1 ^a^	6.3 ^a^	7.1 ^b^	7.5 ^c^	1.02
Firmness (N)	6.94 ^d^	6.12 ^c^	5.92 ^b^	5.04 ^a^	0.98
Stickiness (N)	2.1 ^a^	2.4 ^b^	3.0 ^c^	3.5 ^d^	0.85
*Thermal properties*					
Onset temperature (°C)	60.1 ^a^	61.7 ^b^	62.8 ^c^	63.1 ^c^	1.88
Peak temperature (°C)	66.4 ^a^	67.3 ^b^	67.9 ^c^	68.1 ^c^	2.12
Conclusion temperature (°C)	72.2 ^c^	71.9 ^b^	70.9 ^a^	70.5 ^a^	1.43
Gelatinization enthalpy (J/g dry starch)	5.8 ^c^	5.4 ^b^	5.2 ^a^	5.1 ^a^	0.63

Values with different superscripts within the same line differed at *p* < 0.05. For each recipe, reported values represented the average of the three pasta batches. ^1^ Dry pasta formulated with 0:100 *w*/*w* MT flour–native triticale flour. ^2^ Dry pasta formulated with 25:75 *w*/*w* MT flour–native triticale flour. ^3^ Dry pasta formulated with 50:50 *w*/*w* MT flour–native triticale flour. ^4^ Dry pasta formulated with 75:25 *w*/*w* MT flour–native triticale flour.

**Table 3:** Color changes of spaghetti formulated with increasing levels of malted triticale (MT) flour prior to and after cooking.

	Substitution with MT				√MSE
	CTR ^1^	25-MT ^2^	50-MT ^3^	75-MT ^4^	
*Uncooked samples*					
Lightness L*	67.6 ^d^	64.1 ^c^	60.4 ^b^	58.4 ^a^	2.78
Redness a*	4.4 ^a^	4.9 ^b^	5.8 ^c^	6.4 ^d^	1.64
Yellowness b*	17.4 ^a^	19.3 ^b^	21.2 ^c^	23.3 ^d^	2.32
∆E*	-	5.4	7.1	9.9	
*Samples cooked to optimum*					
Lightness L*	71.3 ^d^	66.6 ^c^	64.1 ^b^	61.3 ^a^	2.66
Redness a*	3.2 ^a^	3.9 ^b^	4.6 ^c^	5.1 ^d^	1.01
Yellowness b*	14.3 ^a^	16.2 ^b^	18.2 ^c^	19.5 ^d^	1.84
∆E*	-	4.9	6.1	6.5	

Values with different superscripts within the same line differed at *p* < 0.05. For each recipe, reported values represented the average of the three pasta batches. ^1^ Dry pasta formulated with 0:100 *w*/*w* MT flour–native triticale flour. ^2^ Dry pasta formulated with 25:75 *w*/*w* MT flour–native triticale flour. ^3^ Dry pasta formulated with 50:50 *w*/*w* MT flour–native triticale flour. ^4^ Dry pasta formulated with 75:25 *w*/*w* MT flour–native triticale flour.

**Table 4:** Rapidly digestible starch (RDS), slowly digestible starch (SDS), resistant starch (RS) content and in vitro protein digestibility (IVPD, % total nitrogen content) of cooked spaghetti formulated with increasing levels of malted triticale (MT) in the recipe.

	Substitution with MT				√MSE
	CTR ^1^	25-MT ^2^	50-MT ^3^	75-MT ^4^	
Available starch (AS)	29.7 ^b^	30.2 ^a^	30.4 ^a^	29.9 ^a^	3.01
Rapidly digestible starch (RDS)	13.4 ^a^	16.1 ^b^	16.8 ^c^	17.8 ^d^	2.12
Slowly digestible starch (SDS)	16.3 ^c^	14.1 ^b^	13.6 ^b^	12.1 ^a^	2.44
RS	3.0 ^c^	2.9 ^b^	2.6 ^a^	2.7 ^a^	1.12
IVPD	89.8 ^a^	90.1 ^a^	89.8 ^a^	92.7 ^b^	3.33

Values with different superscripts within the same line differed at *p* < 0.05. For each recipe, reported values represented the average of the three pasta batches. ^1^ Dry pasta formulated with 0:100 *w*/*w* MT flour–native triticale flour. ^2^ Dry pasta formulated with 25:75 *w*/*w* MT flour–native triticale flour. ^3^ Dry pasta formulated with 50:50 *w*/*w* MT flour–native triticale flour. ^4^ Dry pasta formulated with 75:25 *w*/*w* MT flour–native triticale flour.

## Data Availability

The original contributions presented in the study are included in the article, further inquiries can be directed to the corresponding author.

## References

[1] Zhu F. (2018). Triticale: Nutritional composition and food uses. Food Chem..

[2] Kamanova S., Yermekov Y., Shah K., Mulati A., Liu X., Bulashev B., Toimbayeva D., Ospankulova G. (2023). Review on nutritional benefits of tritical. Czech J. Food Sci..

[3] Pattison A.L., Trethowan R.M. (2013). Characteristics of modern triticale quality: Commercially significant flour traits and cookie quality. Crop. Pasture Sci..

[4] Tohver M., Kann A., Täht R., Mihhalevski A., Hakman J. (2005). Quality of triticale cultivars suitable for growing and bread-making in northern conditions. Food Chem..

[5] Piazza I., Carnevali P., Faccini N., Baronchelli M., Terzi V., Morcia C., Ghizzoni R., Patrone V., Morelli L., Cervini M. (2023). Combining native and malted triticale flours in biscuits: Nutritional and technological implications. Foods.

[6] Fox G.P., Bettenhausen H.M. (2023). Variation in quality of grains used in malting and brewing. Front. Plant Sci..

[7] Benincasa P., Falcinelli B., Lutts S., Stagnari F., Galieni A. (2019). Sprouted grains: A comprehensive review. Nutrients.

[8] Ikram A., Saeed F., Arshad M.U., Afzaal M., Anjum F.M. (2021). Structural and nutritional portrayal of rye-supplemented bread using fourier transform infrared spectroscopy and scanning electron microscopy. Food Sci. Nutr..

[9] Gunathunga C., Senanayake S., Jayasinghe M.A., Brennan C.S., Truong T., Marapana U., Chandrapala J. (2024). Germination Effects on Nutritional Quality: A Comprehensive Review of Selected Cereal and Pulse Changes. J. Food Compos. Anal..

[10] Marti A., Cardone G., Pagani M.A., Casiraghi M.C. (2018). Flour from sprouted wheat as a new ingredient in bread-making. LWT.

[11] Baranzelli J., Kringel D.H., Colussi R., Paiva F.F., Aranha B.C., de Miranda M.Z., Zavareze E.D.R., Dias A.R.G. (2018). Changes in enzymatic activity, technological quality and gamma-aminobutyric acid (GABA) content of wheat flour as affected by germination. LWT.

[12] Grassi S., Cardone G., Bigagnoli D., Marti A. (2018). Monitoring the sprouting process of wheat by non conventional approaches. J. Cereal Sci..

[13] AOAC (2000). Official Methods of Analysis.

[14] AACC International (2010). Approved Methods of Analysis.

[15] Cervini M., Gabrielli M., Spigno G., Giuberti G. (2023). Characterization of Durum-Wheat Pasta Containing Resistant Starch from Debranched Waxy Rice Starch. Foods.

[16] Englyst K.N., Englyst H.N., Hudson G.J., Cole T.J., Cummings J.H. (1999). Rapidly available glucose in foods: An in vitro measurement that reflects the glycemic response. Am. J. Clin. Nutr..

[17] Dodi R., Di Pede G., Scarpa C., Deon V., Dall’Asta M., Scazzina F. (2023). Effect of the Pasta Making Process on Slowly Digestible Starch Content. Foods.

[18] Suo X., Dall’Asta M., Giuberti G., Minucciani M., Wang Z., Vittadini E. (2024). Effect of “shape” on technological properties and nutritional quality of chickpea-corn-rice gluten free pasta. LWT.

[19] Majzoobi M., Wang Z., Teimouri S., Pematilleke N., Brennan C.S., Farahnaky A. (2023). Unlocking the Potential of Sprouted Cereals, Pseudocereals, and Pulses in Combating Malnutrition. Foods.

[20] Yang B., Yin Y., Liu C., Zhao Z., Guo M. (2021). Effect of germination time on the compositional, functional and antioxidant properties of whole wheat malt and its end-use evaluation in cookie-making. Food Chem..

[21] Singh A.K., Rehal J., Kaur A., Jyot G. (2015). Enhancement of attributes of cereals by germination and fermentation: A review. Crit. Rev. Food Sci. Nutr..

[22] Nkhata S.G., Ayua E., Kamau E.H., Shingiro J.B. (2018). Fermentation and germination Improve nutritional value of cereals and legumes through activation of endogenous enzymes. Food Sci. Nutr..

[23] Augustin L.S.A., Aas A.M., Astrup A., Atkinson F.S., Baer-Sinnott S., Barclay A.W., Brand-Miller J.C., Brighenti F., Bullo M., Buyken A.E. (2020). Dietary Fibre Consensus from the International Carbohydrate Quality Consortium (ICQC). Nutrients.

[24] Ramezani F., Pourghazi F., Eslami M., Gholami M., Mohammadian Khonsari N., Ejtahed H.S., Larijani B., Qorbani M. (2024). Dietary fiber intake and all-cause and cause-specific mortality: An updated systematic review and meta-analysis of prospective cohort studies. Clin. Nutr..

[25] Fuad T., Prabhasankar P. (2010). Role of ingredients in pasta product quality: A review on recent developments. Crit. Rev. Food Sci. Nutr..

[26] Xing B., Zhang Z., Zhu M., Teng C., Zou L., Liu R., Zhang L., Yang X., Ren G., Qin P. (2023). The gluten structure, starch digestibility and quality properties of pasta supplemented with native or germinated quinoa flour. Food Chem..

[27] Kömürcü T.C., Bilgiçli N. (2023). Effect of germinated and heat-moisture treated ancient wheat on some quality attributes and bioactive components of noodles. Food Chem..

[28] Marti A., Pagani M.A., Seetharaman K. (2014). Textural attributes of wheat and gluten free pasta. Food Texture Design and Optimizatio.

[29] Liu C., Jiang X., Wang J., Li L., Bian K., Guan E., Zheng X. (2019). Effect of heat-moisture treatment of germinated wheat on the quality of Chinese white salted noodles. Cereal Chem..

[30] Cardone G., D’Incecco P., Pagani M.A., Marti A. (2020). Sprouting improves the bread-making performance of whole wheat flour (*Triticum aestivum* L.). J. Sci. Food Agric..

[31] Manthey F.A., Schorno A.L. (2002). Physical and cooking quality of spaghetti made from whole wheat durum. Cereal Chem..

[32] Diamante G., Peressini D., Simonato M., Anese M. (2019). Effect of continuous cooking on cooking water properties and pasta quality. J. Sci. Food Agric..

[33] Del Nobile M.A., Baiano A., Conte A., Mocci G. (2005). Influence of protein content on spaghetti cooking quality. J. Cereal Sci..

[34] Namir M., Iskander A., Alyamani A., Sayed-Ahmed E.T.A., Saad A.M., Elsahy K., El-Tarabily K.A., Conte-Junior C.A. (2022). Upgrading common wheat pasta by fiber-rich fraction of potato peel byproduct at different particle sizes: Effects on physicochemical, thermal, and sensory properties. Molecules.

[35] Jribi S., Molnàr H., Antal O.T., Adànyi N., Kheriji O., Naàr Z., Debbabi H. (2019). Zinc fortification as a tool for improving sprout hygienic and nutritional quality: A factorial design approach. J. Sci. Food Agric..

[36] Li C., Oh S.G., Lee D.H., Baik H.W., Chung H.J. (2017). Effect of germination on the structures and physicochemical properties of starches from brown rice, oat, sorghum, and millet. Int. J. Biol. Macromol..

[37] Oseguera-Toledo M.E., Contreras-Jiménez B., Hernández-Becerra E., Rodriguez-Garcia M.E. (2020). Physicochemical changes of starch during malting process of sorghum grain. J. Cereal Sci..

[38] Xu M., Jin Z., Simsek S., Hall C., Rao J., Chen B. (2019). Effect of germination on the chemical composition, thermal, pasting, and moisture sorption properties of flours from chickpea, lentil, and yellow pea. Food Chem..

[39] Cimini A., Poliziani A., Morgante L., Moresi M. (2024). Use of malted pulses to formulate gluten-free fresh-egg pasta. Ital. J. Food Sci..

[40] Khoddami A., Mohammadrezaei M., Roberts T.H. (2017). Effects of sorghum malting on colour, major classes of phenolics and individual anthocyanins. Molecules.

[41] Prado R., Gastl M., Becker T. (2021). Aroma and color development during the production of Specialty malts: A review. Compr. Rev. Food Sci. Food Saf..

[42] Englyst K.N., Hudson G.J., Englyst H.N., Meyers R.A. (2000). Starch Analysis in Food. Encyclopedia of Analytical Chemistry.

[43] Bojarczuk A., Skąpska S., Khaneghah A.M., Marszałek K. (2022). Health benefits of resistant starch: A review of the literature. J. Funct. Foods.

[44] EFSA Panel on Dietetic Products, Nutrition and Allergies (NDA Panel) (2011). Scientific Opinion on the substantiation of health claims related to resistant starch and reduction of post-prandial glycaemic responses (ID 681), “digestive health benefits” (ID 682) and “favours a normal colon metabolism” (ID 783) pursuant to Article 13 of Regulation (EC) No 1924/2006. EFSA J..

[45] Wang Y., Zhou X., Xiang X., Miao M. (2022). Association of Slowly Digestible Starch Intake with Reduction of Postprandial Glycemic Response: An Update Meta-Analysis. Foods.

[46] Goux A., Breyton A.-E., Meynier A., Lambert-Porcheron S., Sothier M., Berghe L.V.D., Brack O., Normand S., Disse E., Laville M. (2020). Design and validation of a diet rich in slowly digestible starch for type 2 diabetic patients for significant improvement in glycemic profile. Nutrients.

[47] Dodi R., Bresciani L., Biasini B., Cossu M., Scazzina F., Taddei F., D’Egidio M.G., Dall’Asta M., Martini D. (2021). Traditional and Non-Conventional Pasta-Making Processes: Effect on In Vitro Starch Digestibility. Foods.

[48] Patterson M.A., Maiya M., Stewart M.L. (2020). Resistant starch content in foods commonly consumed in the United States: A narrative review. J. Acad. Nutr. Diet..

[49] Benítez V., Cantera S., Aguilera Y., Mollá E., Esteban R.M., Díaz M.F., Martín-Cabrejas M.A. (2013). Impact of germination on starch, dietary fiber and physicochemical properties in non-conventional legumes. Food Res. Int..

[50] Gallo V., Romano A., Masi P. (2020). Does the presence of fibres affect the microstructure and in vitro starch digestibility of commercial Italian pasta?. Food Struct..

[51] Ohanenye I.C., Tsopmo A., Ejike C.E., Udenigwe C.C. (2020). Germination as a bioprocess for enhancing the quality and nutritional prospects of legume proteins. Trends Food Sci. Technol..

[52] Gallego-Lobillo P., Ferreira-Lazarte A., Hernández-Hernández O., Villamiel M. (2021). In vitro digestion of polysaccharides: InfoGest protocol and use of small intestinal extract from rat. Food Res. Int..

[53] Sharma B., Gujral H.S. (2020). Modifying the dough mixing behavior, protein & starch digestibility and antinutritional profile of minor millets by sprouting. Int. J. Biol. Macromol..

[54] Arribas C., Cabellos B., Sánchez C., Cuadrado C., Guillamón E., Pedrosa M.M. (2017). The impact of extrusion on the nutritional composition, dietary fiber and in vitro digestibility of gluten free snacks based on rice, pea and carob flour blends. Food Funct..

